# Predictive validity of a tool to resolve borderline grades in OSCEs

**DOI:** 10.3205/zma001324

**Published:** 2020-04-15

**Authors:** Rowan Klein Nulend, Peter Harris, Boaz Shulruf

**Affiliations:** 1 University of New South Wales, Office of Medical Education, Sydney, Australia

**Keywords:** OCSE, borderline grades, assessment, medical students

## Abstract

There is inconclusive evidence suggesting which standard setting method yields the highest validity for pass/fail decisions in examinations. The Objective Borderline Method 2 (OBM2) is a decision-making tool for reclassification of borderline grades to clear pass or clear fail grades to resolve examiner uncertainty for high-stakes pass/fail decisions.

This study evaluated the predictive validity of OBM2 pass/fail decisions, using consecutive years’ Objective Structured Clinical Examination (OSCE) results within a medical cohort (n=271) at the University of New South Wales, Australia. OBM2 decisions in one OSCE (n=687) were compared to marks obtained in a subsequent OSCE via independent samples T-tests and analysis of variance (ANOVA). The extent of the relationship between these two variables determines the predictive validity of OBM2 decisions, given that past student grades are capable of predicting future performance.

OBM2 decisions in an initial OSCE were found to have a statistically significant predictive nature for subsequent OSCE marks (p=.005). For initial decisions which reclassified to a pass grade, subsequent OSCE marks were significantly higher than for the cases where initial decisions were reclassified to a fail grade. Stronger associations were identified between related assessment domains/criteria compared to unrelated domains/criteria (Cohen’s d=.469 vs Cohen’s d=.388 respectively).

Through demonstrating the OBM2 decisions’ predictive association across exams there is support for the OBM2’s predictive validity, deeming it a promising method to be used for resolving examiner uncertainty when making pass/fail decisions within OSCEs.

## Zusammenfassung

Es liegen keine eindeutigen Belege dafür vor, welches Standard-Setting-Verfahren die höchste Validität für Entscheidungen über das Bestehen bzw. Nichtbestehen von Prüfungen ergibt. Die Objective Borderline Method 2 (OBM2) ist ein Instrument zur Entscheidungsunterstützung für die Neueinstufung von Grenznoten als eindeutige Bestehens- oder Nichtbestehensnoten. So können Unsicherheiten der Prüfer bei folgenreichen Entscheidungen über das Bestehen bzw. Nichtbestehen beseitigt werden.

In dieser Studie wurde die Vorhersagevalidität von OBM2-basierten Entscheidungen über das Bestehen bzw. Nichtbestehen unter Verwendung der Ergebnisse der Objective Structured Clinical Examination (OSCE) in aufeinanderfolgenden Jahren innerhalb einer Kohorte von Medizinstudenten (N 71) an der University of New South Wales, Australien, geprüft. OBM2-basierte Entscheidungen in einer OSCE-Prüfung (N=687) wurden mit den in einer darauffolgenden OSCE-Prüfung erhaltenen Noten anhand von t-Tests für unabhängige Stichproben und einer Varianzanalyse (ANOVA) verglichen. Der Umfang des Zusammenhangs zwischen diesen beiden Variablen bestimmt die Vorhersagevalidität von OBM2-basierten Entscheidungen, vorausgesetzt, die vorherigen Noten der Studenten lassen Vorhersagen zur zukünftigen Leistung zu.

Es wurde gezeigt, dass durch OBM2-basierte Entscheidungen bei einer ersten OSCE-Prüfung statistisch signifikante Vorhersagen für die nachfolgenden OSCE-Noten (p=0,005) getroffen werden können. In den Fällen, in denen die Noten aus der ersten Prüfung als Bestehensnote neueingestuft wurden, waren die nachfolgenden OSCE-Noten signifikant besser als in den Fällen, in denen die Noten aus der ersten Prüfung als Nichtbestehensnote neueingestuft wurden. Ein stärkerer Zusammenhang wurde für verwandte Bewertungsdomänen/-kriterien im Vergleich zu nicht verwandten Domänen/Kriterien gefunden (Cohens d=0,469 versus Cohens d=0,388).

Der gezeigte prädiktive Zusammenhang der OBM2-basierten Entscheidungen über Prüfungen hinweg stützt die Vorhersagevalidität der OBM2. Sie wird daher als eine vielversprechende Methode zur Beseitigung von Unsicherheiten der Prüfer bei Entscheidungen über das Bestehen bzw. Nichtbestehen von OSCE-Prüfungen betrachtet.

## 1. Introduction

It is important that any decisions arising from assessment strategies used within a medical program are defensible [1], [2], [3]. Subjectivity reduces the defensibility of an examination; to increase objectivity in OSCE settings it is common that a standard setting method is applied [4]. Standard setting methods are applied to define cut-scores which correspond to a minimum level of proficiency/achievement required in an assessment task [4], [5]. 

A broad range of standard setting methods exists; all methods explored in current literature feature some subjectivities and imprecisions, with inconclusive evidence surrounding their efficacy [6], [7]. Most methods require judgements of experts/judges. Although these judgements are made by experts in the field, it is impossible to be entirely objective in such instances [6], [8], [9]. Since there is no gold-standard for standard setting, validating a standard setting is the most challenging issue in standard setting [8]. Previous studies demonstrated that when two or more standard setting methods are applied to the same data set, each delivers a different cut-score [10], [11], [12]. 

Another issue is the definition of a borderline or, as commonly described “minimally competent” student, and the variability of expert opinions in this domain [13]. A borderline result is observed when the examiner is uncertain whether the observed performance reached the clear pass or clear fail level. This may occur when student’s observed performance lies near the expected cut-score which distinguishes between the pass and fail grades [4]. 

To resolve this issue the Objective Borderline Method (OBM) was introduced [10]. The OBM is a standard setting method which uses the concept of redefining borderline marks into either a pass or fail grade; derived from the proportions of pass, borderline and fails yielded by all examinees [14]. This model is based on probability, using proportions of pass/borderline/fail marks. Instead, most standard setting methods allocate a cut-score based on expert opinion or statistical techniques, as is done with the Angoff method and borderline regression method respectively [10].

Since the introduction of the OBM, the Objective Borderline Method 2 (OBM2) has been developed. The OBM2 is not a standard-setting method, as it does not establish a cut-score. The OBM2, instead, is a decision-making tool for reclassification of borderline grades. It uses only two measures; examinee ability and item difficulty, estimated from all assessment marks from an exam, to reclassify the borderline grade as either pass or fail on a case-by-case basis. The OBM2 was found applicable within standard clinical style examination settings to support pass or fail grade decisions in borderline instances [15].

The OBM2 is a probability based method used to replace a borderline mark with either pass of fail mark given to an examinee for each single item [16], [17]. Thus, an examinee may receive any number of borderline marks, from zero to the total number of items in the examination (in the current study it may span between 0 to 54 per student). A borderline mark is a mark given to the examinee when the examiner is unable to determine that a particular skill was performed either at the clear pass or clear fail level [16], [17]. The reclassification of the borderline marks to either pass or fail is determined by the proportions of passes (p), borderline (b) and fail (f) marks yielded by the students using the formula: “OBM index=(p/[b+p])×(b/[f+b])” [16]. The OBM index is calculated twice; once for marks of all items yielded by the student to determine “student ability”, and once for all marks yielded by each item by all students to determine “item difficulty”. Thus, for every borderline mark there are two OBM indices. Then the OBM indices are compared for a given borderline mark. If “student ability”≥ “item difficulty”, the borderline mark is reclassified to a pass. If “student ability”<“item difficulty” the borderline mark is reclassified to a fail. A detailed explanation of the technicality of the OBM2 in presented in previous research [16]. 

In the setting of education, predictive validity is an important subset of criterion validity, as an important goal of examinations is to predict future performance [18]. Current literature indicates that past student grades predict future performance [19]. If the OBM2 could reflect this expectation within a group of students who have all been allocated the same mark (borderline) and had this reclassified to a pass or a fail, it would enhance the OBM2’s validity as a tool to reclassify borderline grades to either “clear pass” or “clear fail” grades. That is, does the OBM2 decision place a borderline student into a group where their future performance corresponds with what is expected from students, based on past grades.

Previous studies have explained the OBM2 tool and have assessed the tool’s defensibility, feasibility, impact on OSCE results and validity [14], [16], [17]. However, these studies used snapshot data which could not provide any indication of the predictive validity of the OBM2 pass/fail decisions [10], [14], [16]. 

## 2. Aim

The aim of this study was to determine to what extent decisions made by the OBM2 predict future performance. This may determine the predictive validity of pass/fail decisions made by the OBM2. To achieve this, the following research question was used: what is the extent of the association between OBM2 decisions in one OSCE with the marks obtained in a subsequent year’s OSCE? 

## 3. Study setting

This study uses data from OSCEs conducted at the University of New South Wales (UNSW) in Sydney, Australia. UNSW medicine is a six year undergraduate program and has OSCEs in second year, third year and sixth year [20]. This study uses data from year 2 OSCE (referred to as Initial) and year 3 OSCE (referred to as Subsequent) examinations of the same cohort, in two consecutive years (2016-2017). The first two years of the UNSW medicine program are primarily theoretical, with weekly alternating 2-hour long clinical skills sessions on-campus and in the hospital being students’ sole clinical practice. Meanwhile, third year students are placed at an allocated hospital daily throughout the year, allowing students substantially more clinical training [17], [18].

The initial examination assesses students (n=271) across three domains; general communication, clinical communication and physical examination, which are split into nine specific assessment criteria within the marking rubric. Therefore, a student is able to achieve up to nine borderline results per OSCE station. The cohort is divided across four separate sites [21]. The subsequent examination (257 students) uses slightly different assessment criteria (see table 1 (Tab. 1)) [21] and is conducted across nine separate sites. 

Both the initial and subsequent OSCEs consist of six separate stations, with different cases and examiners [21]. Each station has one examiner, with a mix of external and university-affiliated examiners. The initial OSCE allows fifteen minutes per station and emphasises assessment of clinical skills, such as clinical communication, physical examination and general communication [21]. The subsequent OSCE allows ten minutes per station and relies on similar clinical skills, as well as case-specificity; meaning thorough underlying clinical knowledge is necessary to perform well in the examination [21]. These subsequent criteria each have equivalents to the three initial domains and can therefore be compared. Both the initial and subsequent OSCEs allow for one re-attempt after a fail grade. Examiners for the subsequent OSCEs were not aware of student grades yielded in the initial OSCE. 

The study comprised data of 271 students who completed the year 2 OSCE in 2016. The year 2 OSCE consists of six stations, in each of which the student is assessed by nine assessment criteria, resulting with 54 marks per student in year 2 OSCE. Each of the assessment criteria focus on one of the three domains; general communication, clinical communication, or physical examination. In total year 2 OSCE yielded 14,634 marks (f=83 [0.6%]; b=687 [4.7%]; p=13864 [94.7%], the p mark includes both “pass” and “distinction” marks). After the application of the OBM2, which replaced the borderline marks with either passes or fails, the marks are summarised (averaged) by the three domains and reported as such. This study however, focussed only on the 687 borderline marks, since only these were modified to either pass of fail. 

## 4. Methods

Hereafter, “OBM2 decisions to reclassify borderline grades to either clear pass or clear fail grades” will be referred to as “decisions”. 

One data set included all initial borderline results for which decisions were made (n=687); the second included all the subsequent marks correlating to each initial decision. For 58 of the 687 initial borderline decisions (14 students), the subsequent OSCE was not attempted in the consecutive year, meaning these subsequent entries were incomplete and were excluded from the analysis. Therefore 629 sets of decisions (257 students) were analysed. During the initial examination, a student can receive a maximum of nine borderline results per OSCE station, as there are nine criteria according to which students are assessed within each station.

The subsequent data consisted of the original marks across 10 assessment criteria prior the application of OBM2 (five each for physical examination and clinical history stations. Assessment criteria for physical-examination-based and history-based stations were paired to create 5 new unified assessment criteria for the subsequent exam (see table 1 (Tab. 1)). This grouping was conducted by three UNSW clinical examination experts, who together decided which criteria assessed similar skills and could therefore be paired together. 

Data analysis compared the initial decision to the subsequent OSCE mark. The initial decision was used as the independent variable such that the results explore the predictive validity of the decisions. Using the original marks (prior the application of the OBM2) for the subsequent OSCE was important in order to avoid any unexpected unrelated impact the OBM2 might have had on the analysis. Therefore the analysis solely compared associations between decisions in the initial OSCEs and the (unmodified) subsequent OSCE marks. 

The analysis was conducted using SPSS [22] starting with independent samples T-tests. Statistical significance was set at p<0.05. First, initial decisions within any initial assessment domain were compared to subsequent marks for any assessment criterion. 

Further analysis explored the relationship between initial decisions per assessment domain and subsequent marks per assessment criterion. Accordingly, the association of initial decisions and subsequent assessment marks both within related domains, and across different domains can be determined. Cohen’s d effect sizes were calculated for each individual factor [23]. 

Analysis of variance (ANOVA) tested between-subject effects to determine whether the station has a confounding effect on the association between initial decisions and subsequent assessment marks. 

## 5. Results

**Figure 1:**
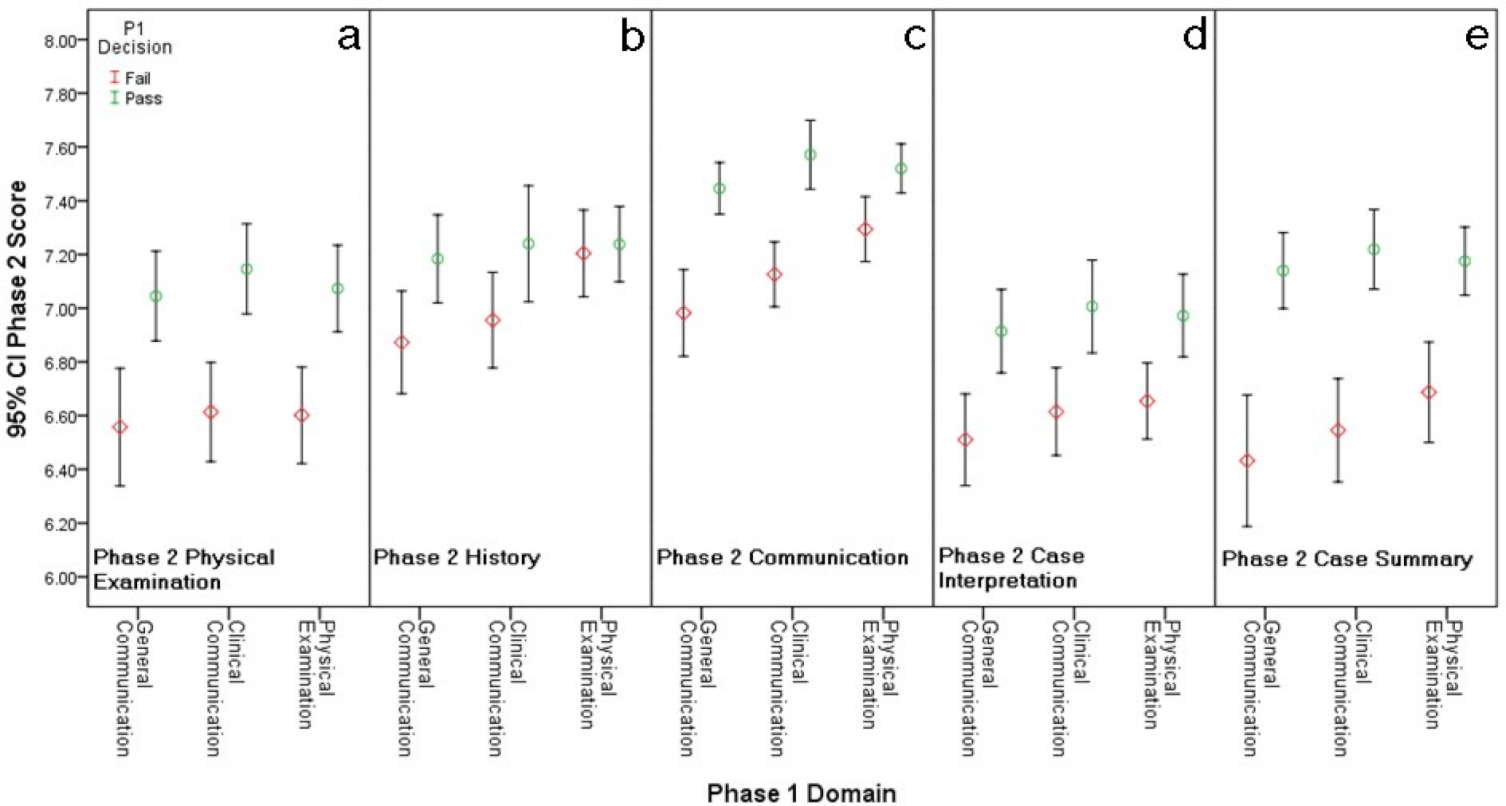
The Association between Initial Decisions per Assessment Domain and Subsequent OSCE Marks per Assessment Criterion

**Figure 2:**
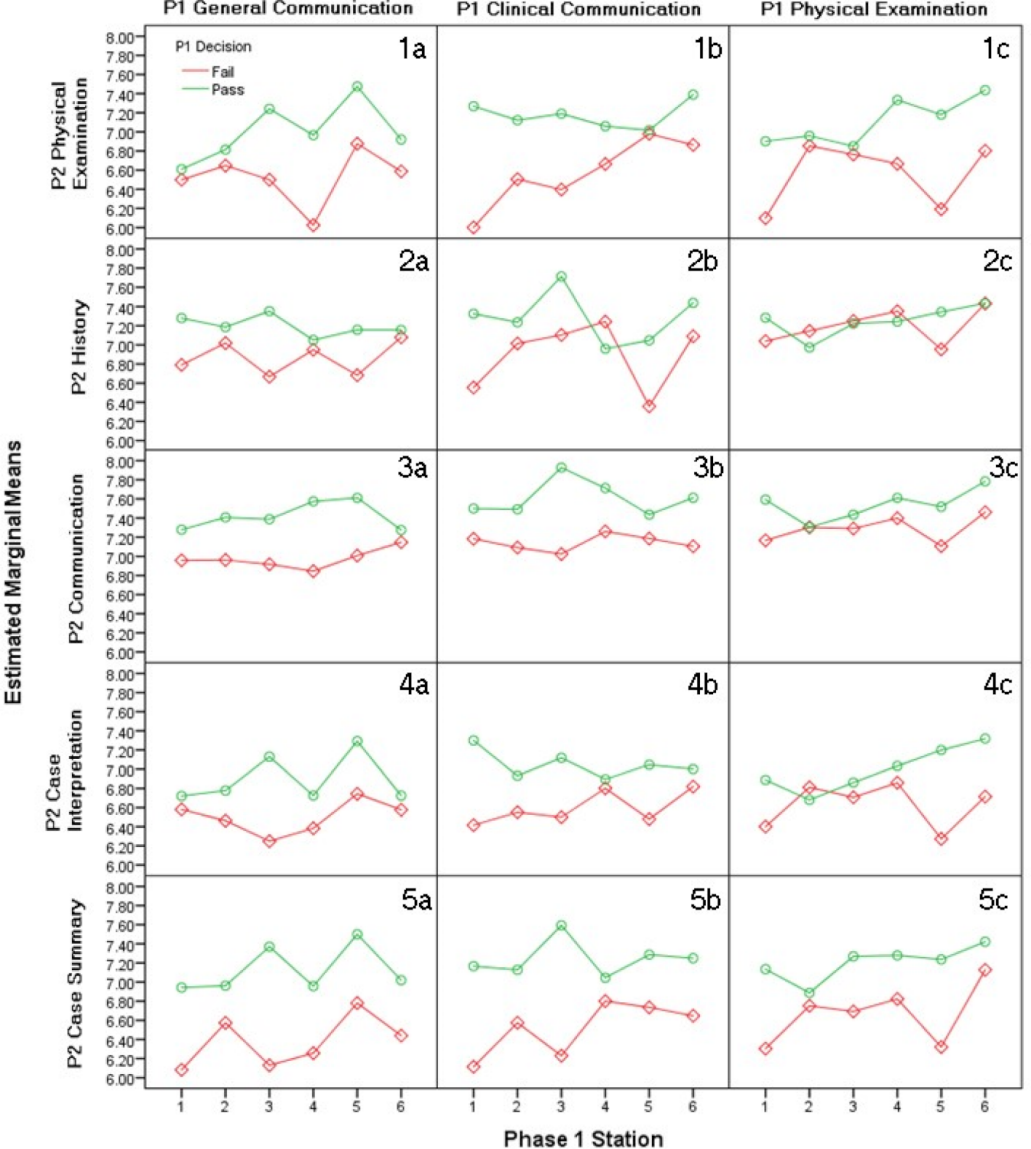
The Predictive Value of an Initial Decision per Assessment Domain for Subsequent OSCE Marks per Assessment Criterion by Station

Independent samples T-tests (see table 2 (Tab. 2) and figure 1 (Fig. 1)) and ANOVA (see figure 2 (Fig. 2)) demonstrate a statistically significant association between the initial decision and the subsequent OSCE performance (examination mark), one year later. 

The T-test demonstrated that across 14 of all 15 comparisons, the subsequent OSCE marks related to initial pass decisions were significantly higher than subsequent OSCE marks related to initial fail decisions (p<.05) (see table 2 (Tab. 2) and figure 2 (Fig. 2)). It is noted that small-medium effect sizes (Cohen’s d=.223-.675) were identified across all the fourteen significant T-tests (see table 2 (Tab. 2)). 

Analysis comparing subsequent OSCE marks to initial decisions within each specific initial assessment domain demonstrated more specific links between initial decisions and subsequent marks (see table 2 (Tab. 2) and figure 1 (Fig. 1)). With one exception; for every assessment domain, initial decisions have a predictive association with every subsequent assessment criterion. The exception is the relationship between decisions made for Initial physical examination, and subsequent history marks (p=.752, Cohen’s d=.041) (see figure 1 (Fig. 1), section b). 

Effect sizes (Cohen’s d) are larger when initial decisions per domain are compared to their related subsequent assessment criteria, than when the comparisons are made across less similar domains (see table 2). Both initial general communication and initial clinical communication have large effects on subsequent communication marks (Cohen’s d=.725 and .691 respectively); furthermore, these two Initial domains have large effects on case summary (Cohen’s d=.708 and .790 respectively) (see table 2 (Tab. 2)). Similarly, initial decisions made for physical examination demonstrated a medium effect on subsequent physical examination marks (Cohen’s d=.506). This also applies for initial physical examination decisions and subsequent case summary marks (Cohen’s d=.558) (see table 2 (Tab. 2)). 

There is a similar statistically significant association in the ANOVA (see figure 2 (Fig. 2)) for each comparison made between related Initial assessment domains and subsequent assessment criteria in independent samples T-tests. 

Initial decisions made in the general communication domain were compared to marks for each subsequent assessment criterion. Similarly, initial decisions made in the general communication and physical examination domains were compared to subsequent marks per assessment criterion. This association again demonstrates that initial pass decisions are associated with significantly higher (p<0.05) subsequent OSCE marks than initial fail decisions; particularly when related domains/criteria. Again, there is no significant association between subsequent history marks and initial decisions in the physical examination domain (see figure 2 (Fig. 2), section 2c). 

Figure 2 (Fig. 2) demonstrates that there is a significant association between the initial decisions and subsequent OSCE scores. There are some outliers (see figure 2 (Fig. 2); sections 2b, 2c, 3c, 4c); however, an overall predictive association exists. Initial pass decisions resulted in consistently higher subsequent marks than initial fail decisions. 

ANOVA determines that this predictive relationship is associated with the initial decision, independent of assessment station. These results indicate that the initial decisions were justified, as past grades should predict future performance, and have managed to do so based on these initial decisions.

## 6. Discussion

Initial decisions have a predictive association when applied to subsequent examinations within a cohort. This predictive validity is stronger within related initial assessment domains and subsequent assessment criteria than across less-related domains/criteria (see table 2 (Tab. 2), see figure 1 (Fig. 1) and figure 2 (Fig. 2)). 

A significant relationship between initial decisions and subsequent OSCE marks exists between initial general and clinical communication decisions, and subsequent history marks (see table 2 (Tab. 2); see figure 1 (Fig. 1), section b; see figure 2 (Fig. 2), section 2a-2b). Whereas, initial decisions in the physical examination domain have no significant association with subsequent history marks (see table 2 (Tab. 2); see figure 1 (Fig. 1), section b; see figure 2 (Fig. 2), section 2c). This is reasonable as the domains assess different skills, whereas communication and history assess similar skills.

Although all three initial assessment domains are significantly associated with subsequent communication marks; initial general and clinical communication decisions acted as substantially stronger predictors than initial physical examination (Cohen's d=.725, .691 and .388 respectively; see table 2 (Tab. 2); see figure 1 (Fig. 1), section c; see figure 2 (Fig. 2), sections 3a-3c). This demonstrates that although the predictive association exists across most domains, it remains strongest within the related domains. 

Due to the requirement for case specificity in the phase 2 OSCEs, case interpretation relies on competent performance within a station to elicit information as well as underlying clinical knowledge to allow discovery and intellectual interpretation of case findings. This is demonstrated by the large effect size related to subsequent OSCE marks in case interpretation and case summary (see table 2 (Tab. 2)). The UNSW Faculty of Medicine specifies that a good case summary relies on multiple factors assessed within the phase 2 OSCE including clear/concise general communication, appropriate clinical jargon, identification of significant case findings and suggestion of differential diagnoses [21]. 

Unmodified grades (borderline) are all identical and are reclassified according to the OBM2 decisions. There is no reason to expect such a predictive association unless decisions are valid. Repeated significant associations throughout different assessment domains/criteria (see figure 2 (Fig. 2)) suggest that this predictability is not a random occurrence. These reclassified grades have a predictive association with future marks; such predictive associations are identified in literature [19]. The ability of decisions to mirror these expectations, especially within related assessment domains/criteria and less-so across unrelated domains/criteria enhances the validity of the decisions. 

Multiple confounders, including the examiner, the examination site and the stations at which the student was examined may have an impact. Each of these is discussed below. 

The UNSW Medicine Faculty uses various organisational strategies to mitigate judgement biases and avoid the occurrence of judgement errors. For the UNSW OSCEs, examiners are randomly selected and allocated to different examination sites. Assessors are rotated between different sites and external assessors are used [24]. Through this process, it is highly unlikely that the same student will be assessed by the same examiner in successive years. 

UNSW data demonstrates that there is no significant difference in OSCE performance between different examination sites [24]. Furthermore, students are randomly allocated to an examination site for each OSCE, thus will not necessarily be assessed at the same site in consecutive years. 

The phase 1 and phase 2 OSCEs are designed to satisfy different syllabi and assess different skills [21]. The OSCE stations at which the student is assessed will not be testing the same skill or clinical knowledge. Therefore, the station at which a student is assessed in the initial OSCE will not alter the association between initial decisions and subsequent OSCE marks. Additionally, ANOVA results establish that there is no significant association between the phase 1 station and phase 2 examination marks for any assessment domain/criterion. 

After excluding each of these variables (examiner, examination site and examination stations), it is evident that most of the predictive nature is related to the decisions.

This provides support for decisions to reclassify borderline grades to clear pass or clear fail grades. The validity of decisions has been asserted through a series of robust statistical tests. In conjunction with previous studies, this report provides further support for the validity of these decisions [7], [14], [17]. Consequently, these decisions resolve examiner uncertainty surrounding borderline scores. This may further increase the objectivity of pass/fail reclassification of borderline marks.

An important limitation is that the study used data from only one cohort of decisions at one university. The study would gain strength and reliability if the same tests were conducted for consecutive years’ OSCE data from different cohorts and across different universities; as well as repetition on this cohort after completion of the third OSCE of the program, or comparison of the OBM2 to other standard setting methods, all of which may be explored in future studies. 

## 7. Conclusion

Decisions have previously been shown to be effective, reliable, defensible and feasible. Previous studies have also suggested that decisions have acceptable validity. This is the first study to demonstrate the predictive validity of decisions, thus further supporting the validity of the decisions. These results may enhance examiners’ confidence when making high-stakes decisions to reclassify borderline grades. 

Further research may establish the OBM2’s unknown limitations. A similar validation study could be repeated when phase 3 OSCE data is available for this cohort (year 2020), to determine whether similar predictive validity is maintained when tested across a third consecutive exam. Furthermore, the OBM2 could be tested within different settings and different examination styles.

## Competing interests

The authors declare that they have no competing interests. 

## Figures and Tables

**Table 1:**
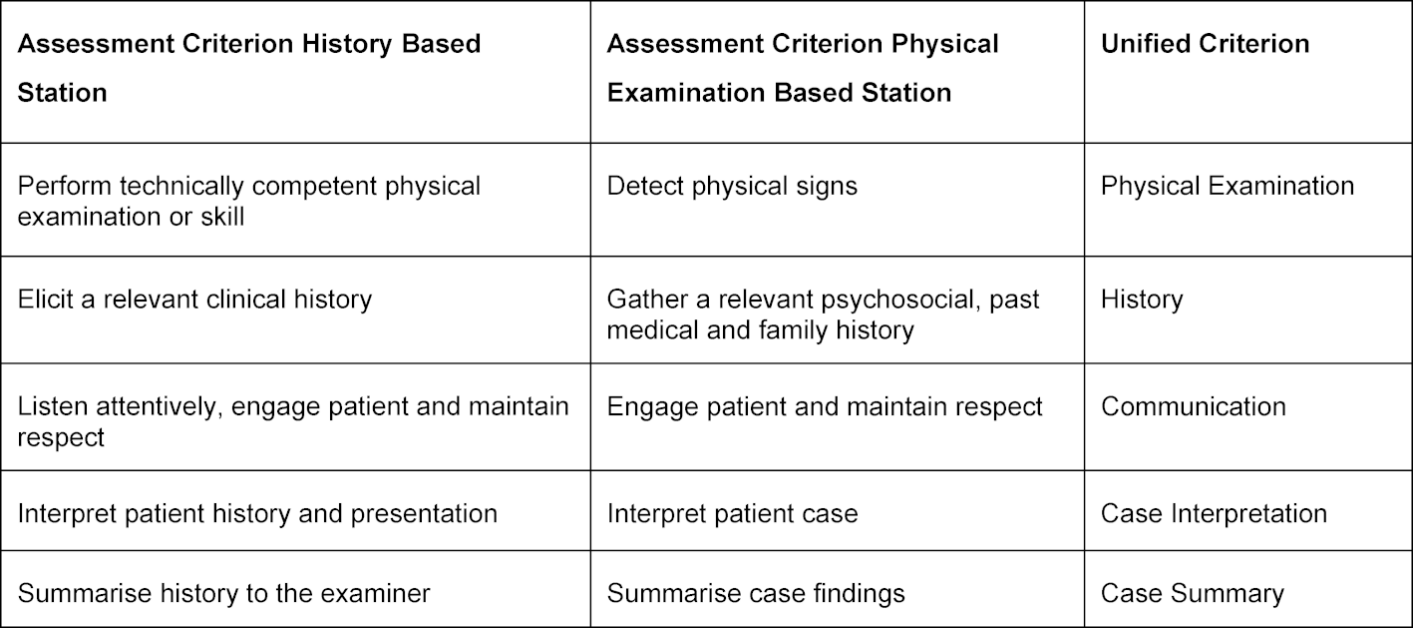
Subsequent Overall Assessment Criteria Generated by Clinical Experts

**Table 2:**
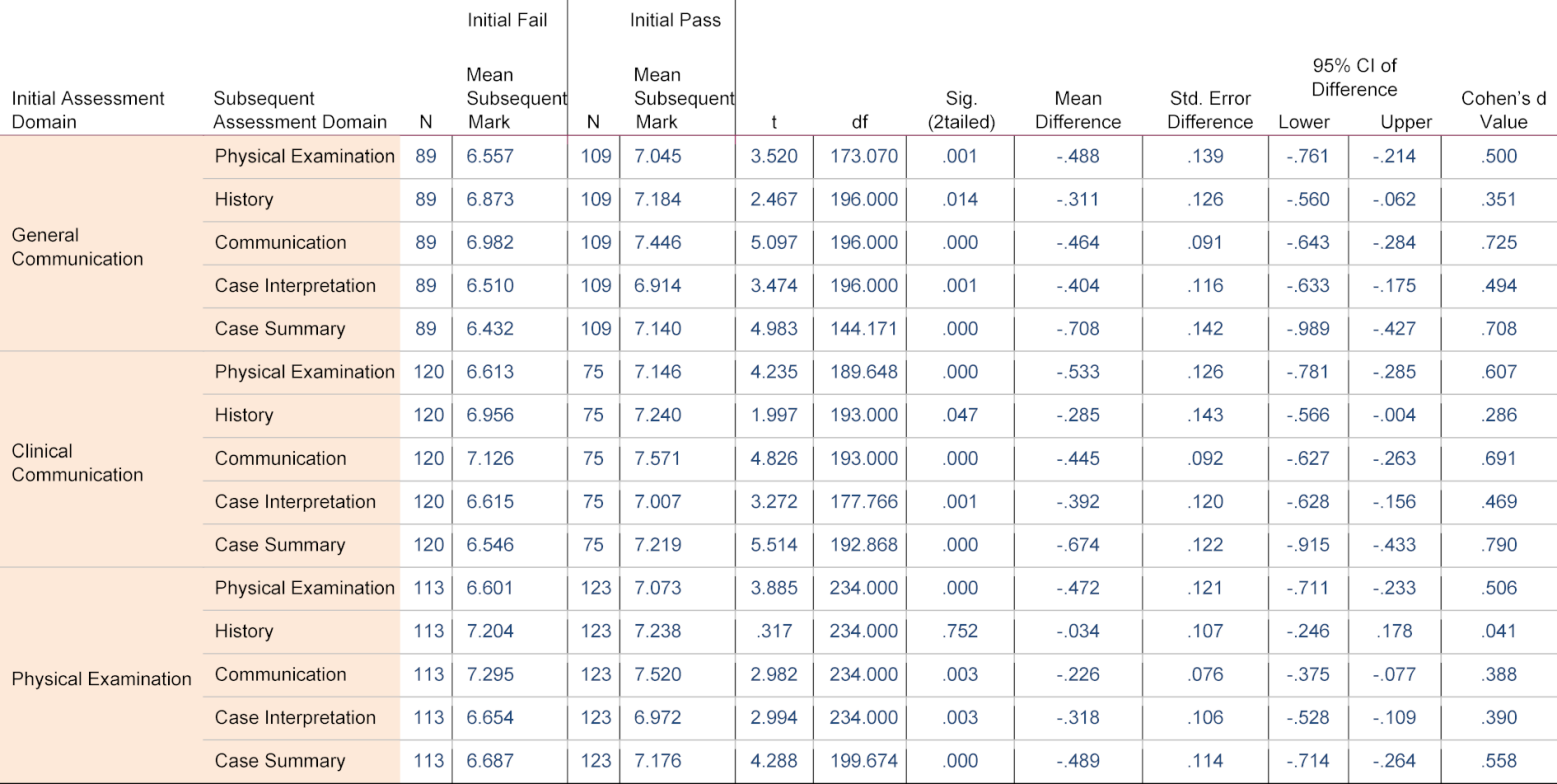
Independent Samples T-Test for the Association between Initial Decisions per Assessment Domain and Subsequent OSCE Marks per Assessment Criterion
